# Peritumoral Brain Edema in Metastases May Be Related to Glymphatic Dysfunction

**DOI:** 10.3389/fonc.2021.725354

**Published:** 2021-10-13

**Authors:** Cheng Hong Toh, Tiing Yee Siow, Mauricio Castillo

**Affiliations:** ^1^ Department of Medical Imaging and Intervention, Chang Gung Memorial Hospital at Linkou, Tao-Yuan, Taiwan; ^2^ Chang Gung University College of Medicine, Tao-Yuan, Taiwan; ^3^ Department of Radiology, University of North Carolina School of Medicine, Chapel Hill, NC, United States

**Keywords:** brain metastasis, peritumoral brain edema, glymphatic system, apparent diffusion coefficient, cerebral blood volume, dynamic susceptibility contrast-enhanced perfusion-weighted imaging, diffusion tensor imaging, ALPS (analysis along perivascular space) index

## Abstract

**Objectives:**

The proliferation of microvessels with increased permeability is thought to be the cause of peritumoral brain edema (PTBE) in metastases. The contribution of the glymphatic system to the formation of PTBE in brain metastases remains unexplored. We aimed to investigate if the PTBE volume of brain metastases is related to glymphatic dysfunction.

**Materials and Methods:**

A total of 56 patients with brain metastases who had preoperative dynamic susceptibility contrast-enhanced perfusion-weighted imaging for calculation of tumor cerebral blood volume (CBV) and diffusion tensor imaging for calculations of tumor apparent diffusion coefficient (ADC), tumor fractional anisotropy (FA), and analysis along perivascular space (ALPS) index were analyzed. The volumes of PTBE, whole tumor, enhancing tumor, and necrotic and hemorrhagic portions were manually measured. Additional information collected for each patient included age, sex, primary cancer, metastasis location and number, and the presence of concurrent infratentorial tumors. Linear regression analyses were performed to identify factors associated with PTBE volume.

**Results:**

Among 56 patients, 45 had solitary metastasis, 24 had right cerebral metastasis, 21 had left cerebral metastasis, 11 had bilateral cerebral metastases, and 11 had concurrent infratentorial metastases. On univariable linear regression analysis, PTBE volume correlated with whole tumor volume (*β* = -0.348, *P* = 0.009), hemorrhagic portion volume (*β* = -0.327, *P* = 0.014), tumor ADC (*β* = 0.530, *P* <.001), and ALPS index (*β* = -0.750, *P* <.001). The associations of PTBE volume with age, sex, tumor location, number of tumors, concurrent infratentorial tumor, enhancing tumor volume, necrotic portion volume, tumor FA, and tumor CBV were not significant. On multivariable linear regression analysis, tumor ADC (*β* = 0.303; *P* = 0.004) and ALPS index (*β* = -0.624; *P* < 0.001) were the two independent factors associated with PTBE volume.

**Conclusion:**

Metastases with higher tumor ADC and lower ALPS index were associated with larger peritumoral brain edema volumes. The higher tumor ADC may be related to increased periarterial water influx into the tumor interstitium, while the lower ALPS index may indicate insufficient fluid clearance. The changes in both tumor ADC and ALPS index may imply glymphatic dysfunction, which is, at least, partially responsible for peritumoral brain edema formation.

## Introduction

Metastases are the most frequent brain tumor in adults (1). Most metastases are associated with peritumoral brain edema (PTBE), which increases intracranial pressure and causes neurological deficits (2). The pathogenesis of PTBE in brain metastases remains unclear and is traditionally thought to represent the net transport of fluid from the intravascular compartment into the brain interstitium due to the proliferation of microvessels which have defects in their inter-endothelial tight junctions (3). This theory, however, does not explain the formation of PTBE in low-grade gliomas with intact tight junctions (4, 5) and meningiomas which are extra-axial and have no direct contact with the brain interstitium.

The glymphatic system has been recently recognized as a pathway for waste clearance and maintaining fluid balance in the brain parenchymal interstitium (6). This highly organized fluid transport system involves cerebrospinal fluid (CSF) inflow along the perivascular spaces of the penetrating arteries and transfer into the brain interstitium under the influence of the aquaporin 4 (AQP4) water channels. With its solute, the CSF–interstitial fluid is then directed towards the venous perivascular spaces, thereafter leaving the brain parenchyma. In rodent experiments, the formation of PTBE has been related to glymphatic dysfunction, including reduced CSF efflux (7) and glymphatic pathway downstream remodeling (8). However, being limited by the invasiveness of current evaluation tools (*e*.*g*., intrathecal contrast medium injection) (9–11), these findings regarding the glymphatic system of animals are not yet confirmed in humans.

Advanced MR imaging techniques, such as dynamic susceptibility contrast-enhanced (DSC) perfusion-weighted imaging (PWI) (12–14) and diffusion tensor imaging (DTI) (12, 15), offer an opportunity for the noninvasive assessment of fluid dynamics in the tumor intravascular compartment, tumor interstitium, and glymphatic system. DSC-PWI dynamically measures T2*-weighted signal intensity loss related to intravascular gadolinium concentration, from which relative cerebral blood volume (CBV) can be computed for the measurement of intravascular fluid volume that is related to microvascular proliferation in tumors (16–19). Apparent diffusion coefficient (ADC) and fractional anisotropy (FA) are quantitative metrics derived from DTI for water diffusivity measurement. In addition to tumor cellularity (20) and fluid viscosity (21), they may also reflect the volume and flow directionality of the tumor interstitial fluid (12). The ALPS index, recently proposed by Taoka et al., is another quantitative diffusion metric derived from DTI (22). It estimates the diffusivity along the perivascular spaces of medullary veins and has been used as a noninvasive quantitative marker to assess human glymphatic activity in clinical conditions including Alzheimer’s disease (22, 23), normal pressure hydrocephalus (24, 25), Parkinson disease (26, 27), age-related iron deposition (28), diabetic cognitive impairment (29), and meningioma-associated brain edema (30).

To the best our knowledge, the associations of PTBE volume with tumor diffusion and perfusion properties and glymphatic function in patients with brain metastasis remained unexplored. In this study, we took advantage of these advanced MR techniques to evaluate the changes in fluid dynamics associated with brain metastases. We hypothesized that the PTBE of metastases is associated with fluid dynamics in the tumor intravascular compartment, tumor interstitium, and glymphatic system as evidenced by alterations of tumor CBV, tumor ADC, tumor FA, and ALPS index.

## Materials and Methods

### Study Subjects

Approval for reviewing the clinical data of the patients and the preoperative MRI studies was obtained from our institutional review board. Between 2006 and 2018, a total of 74 consecutive patients with subsequent histopathological diagnosis of brain metastasis underwent preoperative MRI using a dedicated tumor protocol which included DSC-PWI and DTI at our institution. These patients were initially screened for eligibility to enter prospective glioblastoma studies but were later excluded due to a pathologic diagnosis of brain metastasis. A total of 18 patients were excluded due to motion artifacts (*n* = 2), purely hemorrhagic tumors (*n* = 14), and tumors limited to the infratentorial compartment (*n* = 2). Patients with partial hemorrhagic tumors were included if their enhancing tumor portions were not obscured by susceptibility artifacts.

Thus, a total of 56 patients (30 women, 26 men; mean age, 56.9 ± 11.6 years; age range, 34–77 years) were analyzed. No patients had begun corticosteroid treatment, diuretic therapy, radiation therapy, and chemotherapy or had a previous brain surgery at the time of their MRI studies. An overview of the characteristics of the patients is found in **Table 1**.

**Table 1:** Patient characteristics.

Characteristics	Number of patients
Number of patients	56
Mean age ± SD (years)	56.9 ± 11.6
Sex	
Woman	30
Man	26
Cerebral hemisphere involved	
Right	24
Left	21
Bilateral	11
Tumor location	
Frontal	24
Parietal	12
Occipital	9
Temporal	7
Deep gray nucleus	4
Number of tumors	
Solitary	45
Multiple	11
Concurrent infratentorial tumors	
No	45
Yes	11
Primary cancer	
Lung	32
Breast	7
Genitourinary system	6
Gastrointestinal tract	4
Liver	2
Head and neck	1
Unknown primary	4

Except where indicated, data are numbers of patients.

SD, standard deviation.

### Clinical and Imaging Information

The medical records of patients and MRI studies were retrospectively reviewed to collect clinical and imaging information, including sex, age, primary cancer, cerebral hemisphere involved (right, left, or bilateral cerebral hemispheres), tumor location, number of tumors (solitary or multiple), and concurrent infratentorial tumors (yes or no). A histopathologic diagnosis was made by a board-certified neuropathologist with 20 years of experience.

### MRI

All MRI studies were performed using a 3-T unit (Magnetom Tim Trio, Siemens, Erlangen, Germany) with a 12-channel phased-array head coil. All examinations included T2-weighted, DSC-PWI, DTI, and T1-weighted sequences acquired in the transverse plane before and after administration of the gadolinium contrast medium. DTI was performed using single-shot echo-planar imaging with the following parameters: TR ms/TE ms, 5,800/83; diffusion gradient encoding in 20 directions; *b* = 0, 1000 s/mm^2^; field of view (FOV), 256 × 256 mm; matrix size, 128 × 128; section thickness, 2 mm; and number of signals acquired, four. A total of 50–60 sections without intersection gap were used to cover the cerebral hemispheres, brainstem, and upper cerebellum. Generalized autocalibrating partially parallel acquisitions with the reduction factor set at 2 were used during DTI acquisitions.

DSC-PWI was obtained with a T2*-weighted gradient-echo EPI sequence during a bolus injection of a standard dose (0.1 mmol/kg) of intravenous gadopentetate dimeglumine (Magnevist; Schering, Berlin, Germany). The injection rate was 4 ml/s for all patients and was immediately followed by a bolus injection of saline (total of 20 ml at the same rate). The DSC-PWI sequence parameters included the following: TR/TE, 1,640/40 ms; flip angle, 90°; FOV, 230 × 230 mm; section thickness, 4 mm; 20 sections and acquisition time of 1 min and 28 sec. A total of 50 measurements were acquired, allowing at least five measurements before bolus arrival. No contrast agent was administered before DSC-PWI. Contrast-enhanced T1-weighted images (TR/TE, 2,000/2.63 ms; section thickness, 1 mm; TI, 900 ms; acquisition matrix, 224 × 256, and FOV, 224 × 256 mm) were acquired after completion of the DTI and DSC-PWI sequences.

### Image Postprocessing and Analysis

The software nordic Image Control and Evaluation, version 2 (Nordic Imaging Lab, Bergen, Norway), was used for all volume measurements and for processing of perfusion and diffusion tensor data. All images were coregistered based on a 3D non-rigid transformation and mutual information. The adequacy of registration was visually assessed, and manual adjustments were performed by changing the transformation parameters of translation, rotation, and/or scaling as necessary. The ALPS index was measured with 3D Slicer, version 4.10.2 (http://www.slicer.org). Two neuroradiologists (with 16 and 6 years of experience, respectively) independently performed all measurements. If the tumors were found in both hemispheres, only those in the hemisphere with a larger PTBE volume were selected for measurements of volume, perfusion, and diffusion metrics. If multiple tumors or PTBE areas were present, all were included as long as their sizes were larger than 1 × 1 cm^2^.

### Measurements of Volume of PTBE, Whole Tumor, Enhancing Tumor Portions, Necrotic Portions, and Hemorrhagic Portions

**Figure 1:**
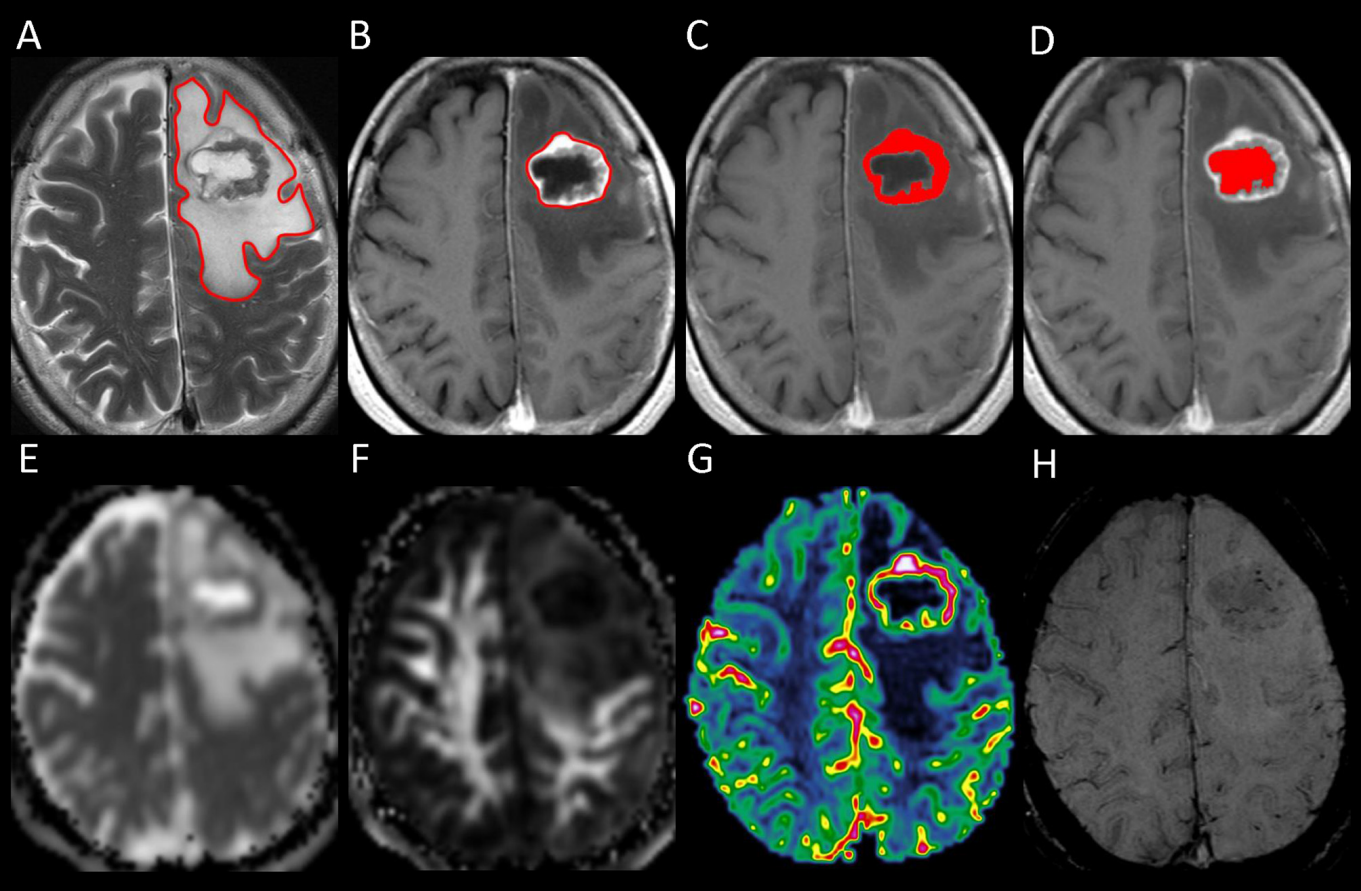
Example of how regions of interest (ROIs) were segmented in a left frontal metastatic brain tumor. Transverse T2-weighted image **(A)** shows a manually drawn polygonal ROI that includes the entire peritumoral brain edema and the whole tumor. On contrast-enhanced T1-weighted images **(B–D)**, the ROIs of whole tumor **(B)**, enhancing tumor **(C)**, and necrotic portion **(D)** are shown. The ROI of the enhancing tumor **(C)** is used to measure the tumor apparent diffusion coefficient (ADC), tumor fractional anisotropy (FA), and tumor cerebral blood volume (CBV) by overlaying the ROI on the corresponding ADC **(E)**, FA **(F),** and CBV **(G)** maps. On susceptibility-weighted image **(H)**, there is no hemorrhagic tumor portion.

One polygonal region of interest (ROI) was first placed on each T2-weighted image to include the entire PTBE and tumor, followed by another ROI drawn to include the entire tumor on each contrast-enhanced T1-weighted image. Subtracting the second ROI from the first ROI yielded the isolated PTBE area. If necrotic and hemorrhagic portions were present, they were measured by placing the ROIs on contrast-enhanced T1- and T2-weighted images, respectively, with reference to precontrast T1-weighted and SWI images. Subtracting ROIs of necrotic and hemorrhagic portions from tumor ROI yielded the enhancing tumor area. The slice volume of each ROI was computed by multiplying the area by slice distance (slice thickness + slice gap). The total volumes of PTBE, whole tumor, enhancing tumor portion, necrotic portion, and hemorrhagic portion were calculated by summing up all slice volumes. An example of ROI segmentation is shown in **Figure 1**.

### Measurements of ADC, FA, and CBV of Enhancing Tumor Portions

Diffusion-weighted images were co-registered to the non-diffusion weighted (*b* = 0) images to minimize artifacts induced by eddy current and subject motion. The ADC and FA were calculated from diffusion tensor data using standard algorithms (12, 15). The CBV for each voxel was estimated by integrating the relaxivity–time curve converted from the dynamic signal intensity curve. Contrast leakage correction was performed as it has been shown to improve tumor grading by using a technique outlined by Boxerman et al. (13, 14).

The ADC, FA, and CBV of enhancing tumor were measured by using the ROIs transformed from contrast-enhanced T1-weighted space. The mean ADC, FA, and CBV values of the whole enhancing tumor volume were calculated by averaging the values of all slices, with the enhancing tumor volume of each slice taken into account. Before all quantitative comparisons, the mean CBV values were normalized and expressed as ratios. The ratios were calculated by dividing the mean values of the whole tumor by the values obtained from a circular ROI (size range, 50–100 mm^2^) placed in the contralateral normal-appearing white matter.

### Measurement of ALPS Index

DTI-ALPS method (22) was used to evaluate the glymphatic function. This method evaluates the diffusivity along the perivascular space on a transverse slice at the level of the lateral ventricle body. The medullary veins, accompanied by their perivascular spaces, run perpendicular to the ventricular walls at the level of the lateral ventricular bodies in a right–left or left–right direction (*i*.*e*., *x*-axis in the image coordinates). In this level, the corticofugal corona radiata projection fibers run in the craniocaudal direction (*i*.*e*., *z*-axis in the image coordinates) adjacent to the lateral ventricles. The superior longitudinal fascicle, which represents the association fibers, runs in the anterior–posterior direction (*i*.*e*., *y*-axis in the image coordinates) and is located lateral to the corona radiata. As the perivascular space is nearly perpendicular to both the projection fibers and association fibers, the major difference between the *x*-axis diffusivity in both fibers (*D*_xproj_ and *D*_xassoc_ for *x*-axis diffusivity in projection fiber and association fiber, respectively) and the diffusivity that is perpendicular to the *x*-axis and to the direction of fiber tracts (*y*-axis for projection fiber, where diffusivity is denoted as *D*_yproj_; *z*-axis for association fiber, where diffusivity is denoted as *D*_zassoc_) is the existence of the perivascular space. To quantify glymphatic activity, the ALPS index is defined as follows:


(1) $$\text{ALPS index }=\frac{\text{mean}(D_{\text{xproj}},D_{\text{xassoc}})}{\text{mean}(D_{\text{yproj}},D_{\text{zassoc}})}$$

**Figure 2:**
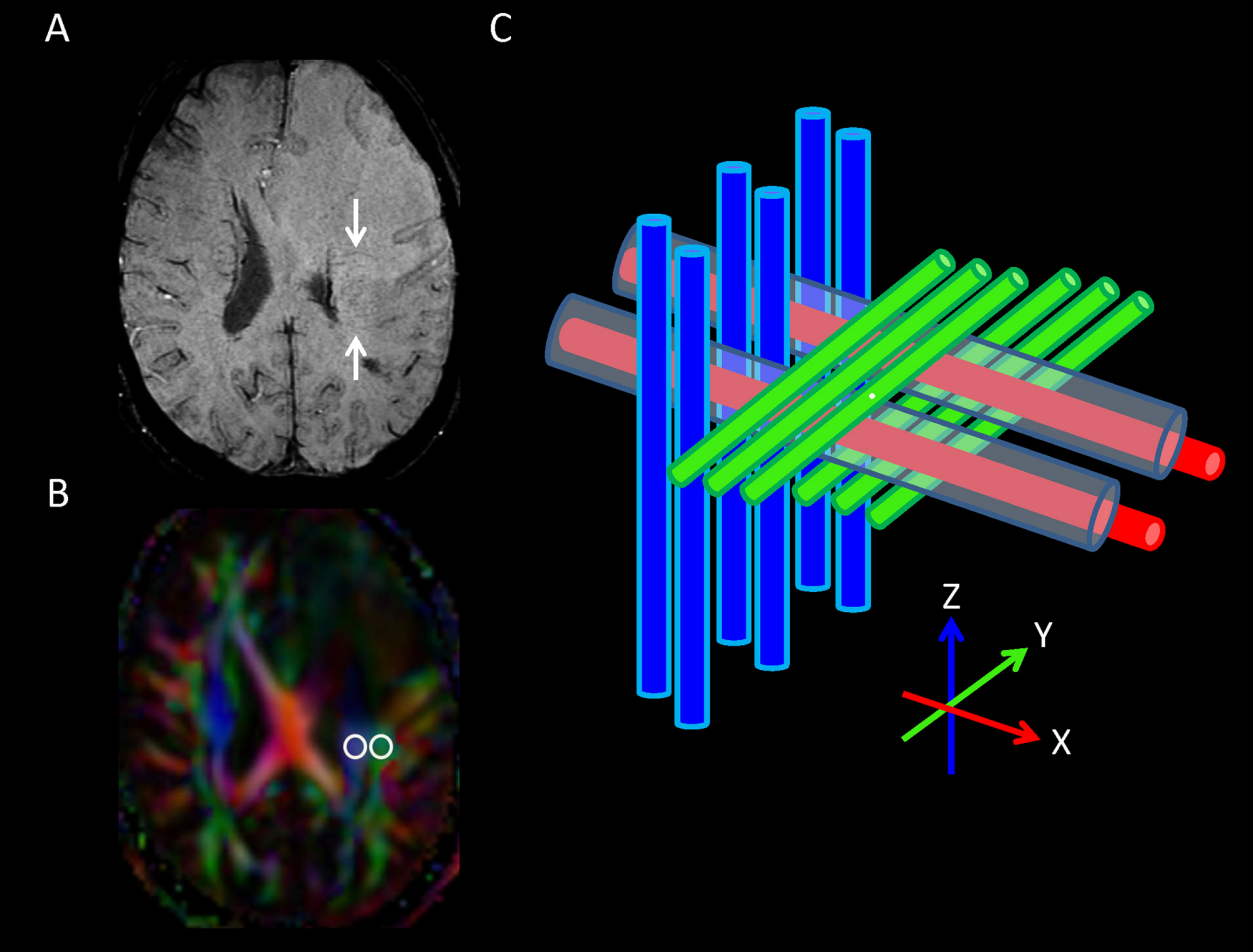
**(A)** On transverse susceptibility-weighted image, the deep medullary veins (arrows) run in the right–left direction (x-axis) at the level of the lateral ventricle body. **(B)** Directionally encoded color map illustrates the regions of interest of projection (blue area) and association (green area) fibers in the left periventricular region for calculation of analysis along the perivascular space ALPS index. **(C)** Schematic diagram presenting the relationship between the directions of the perivascular spaces (gray cylinders) surrounding the deep medullary veins (red cylinders; x-axis), the projection fibers (blue cylinders; z-axis), and the association fibers (green cylinders; y-axis). Note that the direction of the perivascular spaces is perpendicular to both the projection and association fibers.

Diffusion metric images were generated by using 3D Slicer, version 4.10.2 (http://www.slicer.org). The ROIs of projection (mean size, 35 ± 19 mm^2^) and association fibers (mean size, 30 ± 18 mm^2^) were drawn on a slice at the level of the lateral ventricular body based on a directionality encoded map. The ALPS index was computed according to the equation above (1). The concept of DTI-ALPS method and an example of ROI placement for ALPS index measurement are shown in **Figure 2**.

### Statistical Analysis

A commercially available statistical software package (SPSS 22; IBM, Armonk, New York) was used for analysis, and *P*-values <0.05 were considered to indicate a statistical significance. Continuous variables are denoted as mean ± standard deviation unless otherwise noted. The Kolmogorov–Smirnov test was used to assess the normality of continuous variables and guide the selection of a parametric or nonparametric test for the comparison of variables. Variance inflation factors were used to detect multicollinearity.

The interobserver variability in the measurements of volumes, ADC, FA, CBV, and ALPS index was assessed by intraclass correlation coefficients (ICCs) with 95% confidence intervals based on an absolute‐agreement, two‐way, random‐effects model. The final values of all measurements were obtained by taking the mean of the independent measurements of two observers.

The associations of PTBE volume with age, sex, tumor location, cerebral hemisphere involved, number of tumors, concurrent infratentorial tumors, primary cancer (lung cancer *vs*. others), whole tumor volume, enhancing tumor volume, necrotic portion volume, hemorrhagic portion volume, tumor ADC, tumor FA, tumor CBV, and ALPS index were first analyzed with univariable linear regression. All variables were entered as potential covariates in the stepwise multivariable linear regression analysis to identify independent factors associated with PTBE volume.

## Results

Among 56 patients, 45 had solitary metastasis, 24 had right cerebral metastases, 21 had left cerebral metastases, 11 had bilateral cerebral metastases, and 11 had concurrent infratentorial metastases. The locations of the metastatic tumors were frontal (*n* = 24), parietal (*n* = 12), occipital (*n* = 9), temporal (*n* = 7), and deep gray nucleus (*n* = 4). The primary sites of tumors were the lung (*n* = 32), breast (*n* = 7), genitourinary (*n* = 6), gastrointestinal (*n* = 4), liver (*n* = 2), head and neck (*n* = 1), and unknown (*n* = 4) primaries. **Table 1** depicts the clinical characteristics of 56 patients.

There were excellent interobserver agreements (ICC = 0.084–0.998, *P* < 0.001) in the measurements of PTBE volumes, whole tumor volumes, enhancing tumor volumes, necrotic portion volumes, hemorrhagic portions volumes, tumor ADC, tumor FA, tumor CBV, and ALPS index. The mean volumes (cm^3^) of PTBE, whole tumor, enhancing tumor, necrotic portion, and hemorrhagic portion were 87.67 ± 45.77, 24.95 ± 21.56, 17.80 ± 16.34, 2.53 ± 5.74, and 4.61 ± 11.37, respectively. The mean tumor ADC (10^-6^ mm^2^/s), tumor FA, tumor CBV, and ALPS index were 1091.8 ± 195.2, 0.08 ± 0.03, 7.57 ± 5.28, and 1.226 ± 0.176, respectively. **Table 2** summarizes all measurements.

**Table 2:** Results of volume, ADC, FA, CBV, and ALPS index measurements.

Measurement	Mean ± SD	Range
Peritumoral edema volume (cm^3^)	87.67 ± 45.77	0–178.09
Whole tumor volume (cm^3^)	24.95 ± 21.56	2.64–85.22
Enhancing tumor volume (cm^3^)	17.80 ± 16.34	1.15–72.11
Necrotic portion volume (cm^3^)	2.53 ± 5.74	0–36.72
Hemorrhagic portion volume (cm^3^)	4.61 ± 11.37	0–49.40
Tumor ADC ± SD (10^-6^ mm^2^/s)	1,091.8 ± 195.2	763.0–1,606.9
Tumor FA ± SD	0.08 ± 0.03	0.04–0.19
Tumor CBV ratio ± SD	7.57 ± 5.28	0.28–21.64
Mean ALPS index ± SD	1.226 ± 0.176	0.910–1.612

ADC, apparent diffusion coefficient; ALPS, analysis along the perivascular space; CBV, cerebral blood volume; FA, fractional anisotropy; SD, standard deviation.

**Figure 3:**
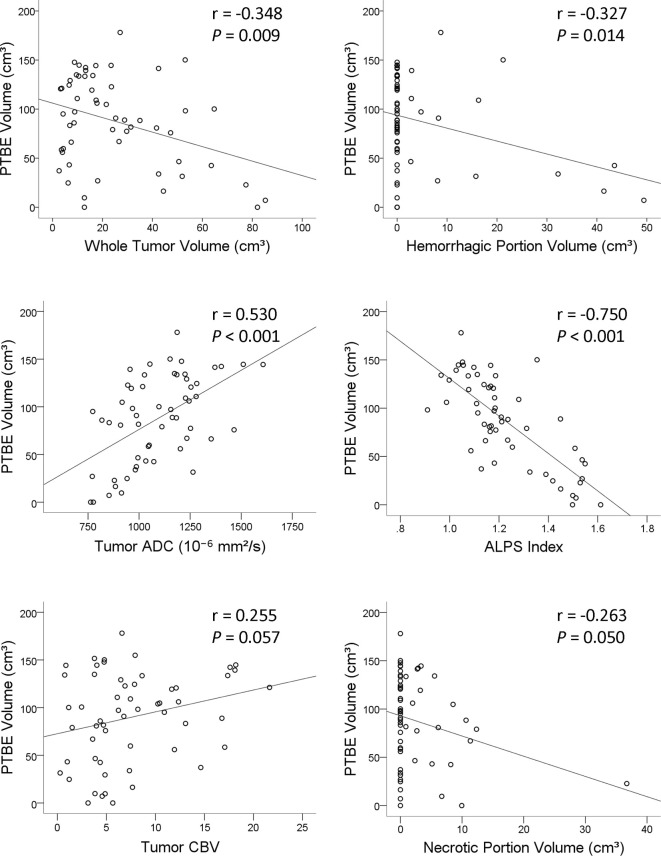
Scatterplots with regression line showing the correlations of the peritumoral brain edema volume of metastatic brain tumors with whole tumor volume, hemorrhagic portion volume, tumor apparent diffusion coefficient, analysis along the perivascular space index, tumor cerebral blood volume, and necrotic portion volume.

On univariable linear regression analysis, the PTBE volume correlated with whole tumor volume (*β* = -0.348, *P* = 0.009), hemorrhagic portion volume (*β* = -0.327, *P* = 0.014), tumor ADC (*β* = 0.530, *P* < 0.001), and ALPS index (β = -0.750, *P* < 0.001). The associations of PTBE volume with necrotic portion volume (*β* = -0.263, *P* = 0.050) and tumor CBV (*β* = 0.255, *P* = 0.057) were marginally significant. **Figure 3** shows the correlations of PTBE volume with factors that were significant or approaching significance on univariable linear regression. No correlations were found between PTBE volume and age, sex, tumor location, number of tumors, enhancing tumor volume, concurrent infratentorial tumor, and tumor FA.

**Figure 4:**
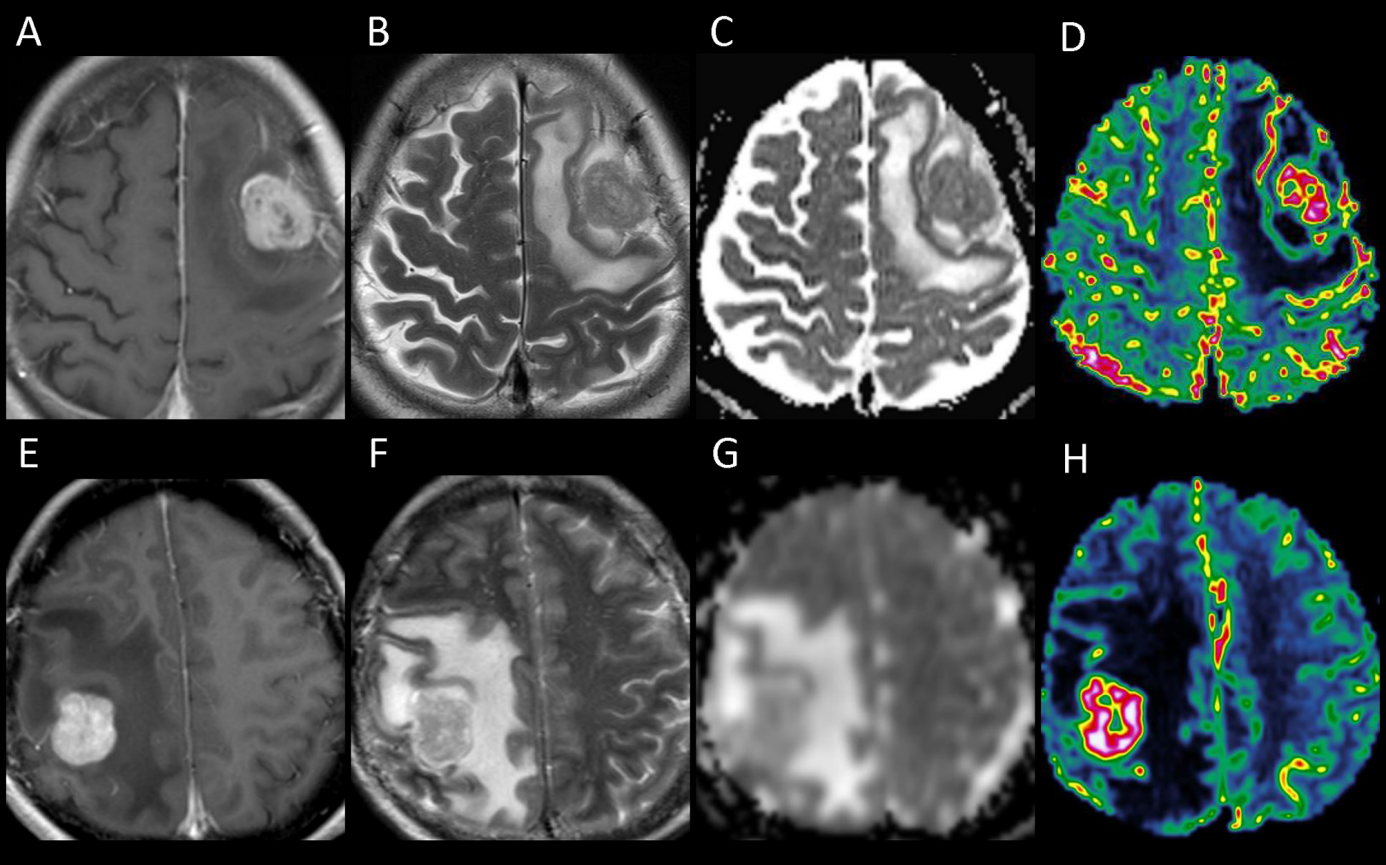
The upper panel shows the contrast-enhanced T1-weighted **(A)**, T2-weighted **(B)**, apparent diffusion coefficient (ADC) **(C)**, and cerebral blood volume (CBV) **(D)** images of a left frontal metastasis with peritumoral brain edema (PTBE) of 85.98 cm^3^, ADC value of 817.9 × 10^-6^ mm^2^/s, and relative CBV of 4.37. The lower panel **(E–H)** shows the corresponding images from a right parietal metastasis with PTBE of 133.63 cm^3^, ADC value of 1,185 × 10^-6^ mm^2^/s, and relative CBV of 17.37. The ADC and CBV of the right parietal metastasis with a larger PTBE volume are higher than those of the left frontal metastasis which has a smaller PTBE volume.

On stepwise multiple linear regression analysis, tumor ADC (*β* = 0.303; *P* = 0.004) and ALPS index (*β* = -0.624; *P* < 0.001) were the two independent factors associated with PTBE volume. The results of univariable and multivariable linear regression analyses of factors associated with PTBE volume are summarized in **Table 3**. Examples of brain metastases with small and large volumes of PTBE are shown in **Figure 4**.

**Table 3:** Univariable and multivariable linear regression analyses of factors associated with peritumoral brain edema (PTBE) volume.

Characteristics	PTBE volume							
	Univariable linear regression				Multivariable linear regression			
	B	SE	β	*P-*value	B	SE	β	*P-*value
Age	0.665	0.527	0.169	0.213				
Sex	4.426	12.363	0.049	0.722				
Cerebral hemisphere involved	9.237	8.071	0.154	0.257				
Tumor location	1.558	4.935	0.043	0.754				
Number of tumors	-3.372	15.529	-0.033	0.811				
Concurrent infratentorial tumor	-2.308	15.534	-0.020	0.882				
Primary cancer	13.052	12.347	0.142	0.295				
Whole tumor volume	-0.739	0.271	-0.348	0.009*				
Enhancing tumor volume	-0.389	0.377	-0.139	0.307				
Necrotic portion volume	-2.093	1.046	-0.263	0.050				
Hemorrhagic portion volume	-1.316	0.517	-0.327	0.014*				
Tumor ADC	1.242	0.271	0.530	<.001*	0.723	0.236	0.303	0.004
Tumor FA	-160.961	201.864	-0.108	0.429				
Tumor CBV	2.290	1.180	0.255	0.057				
ALPS Index	-193.578	23.482	-0.750	<.001*	-167.747	26.493	-0.624	<.001

ADC, apparent diffusion coefficient; ALPS, analysis along the perivascular space; CBV, cerebral blood volume; FA, fractional anisotropy; SD, standard deviation; B, unstandardized coefficient; β, standardized coefficient; SE, standard error.

*P-value <0.05.

## Discussion

Our study showed that the PTBE volume of metastases correlated positively with tumor ADC and inversely with ALPS index. Metastases with a larger volume of PTBE had higher ADC and lower ALPS index. These findings suggest that the PTBE of metastases may be related to intratumoral water diffusivity and glymphatic dysfunction. In contrast, changes of tumor intravascular fluid volume may not contribute to PTBE formation as the tumor CBV was not correlated with the PTBE volume.

Metastatic brain tumors are known to have a disrupted inter-endothelial tight junction (31) due to the downregulation of tight junction components, including claudin-1, claudin-5, and occludin (32, 33). With the increased microvascular permeability, intravascular fluid has been considered as the water source of PTBE. Tumor CBV is a surrogate marker of angiogenesis as it correlates with microvascular proliferation (16–19) and the expression of endothelial growth factor (34, 35). In our study, the association between tumor CBV and PTBE volume was not significant, suggesting that the proliferation of microvessels, and thus the increased intravascular fluid volume, is not related to PTBE formation. This finding agrees with the results of a previous study in which no correlations were present between the PTBE volume of brain metastases and microvessel density as determined by anti-CD34 staining (36). Based on these findings, we speculate that intravascular fluid may not be the water source of PTBE.

Systemic steroids are the mainstay of treatment for PTBE (37) and may result in rapid edema reduction and symptom relief through the restoration of tight junctions and reduction of capillary permeability by binding to glucocorticoid receptors (38). This effect, however, is typically transient and diminishes within weeks or months (39), suggesting that PTBE formation may be related to causes other than the disruption of tight junctions. A recent study shows that the blood–brain barrier is more complex than anticipated (40). Changes in the supporting structures of the blood–brain barrier, such as astrocyte, pericytes, and microglial cells, may also be associated with influx of fluid into the brain interstitium. The astrocyte covering of brain microvessels seems to be rate limiting to water movement (40), and it is suggested that water channels AQP4 located on astrocytic foot processes may play a significant role in PTBE formation. A strong correlation between PTBE and upregulated astrocyte AQP4 expression in human astrocytomas and metastatic adenocarcinomas suggests that increased AQP4 expression may be essential to the pathogenesis of PTBE (41). Since AQP4 water channels are part of the glymphatic system, we also postulate the possibility that PTBE in metastases may be related to glymphatic dysfunction with an increased periarterial influx of CSF into the tumor interstitium. While the current understanding of the mechanism of PTBE was developed prior to the discovery of the glymphatic system, incorporating the role of the glymphatic system into the current theory of PTBE formation may help in the development of effective treatments for reducing PTBE.

Although ADC has been considered as a marker of tumor cellularity, the correlations between the two were inconsistent (42). As ADC measures extracellular water diffusivity and the quantity of mobile water molecules, it may also reflect fluid volume in the tumor interstitium. In our study, tumors with higher ADC were associated with a larger PTBE volume. We hypothesize that the higher tumor ADC may reflect water increase in the tumor interstitium as a consequence of AQP4-mediated fluid influx. On the other hand, a correlation between AQP4 expression and ADC changes was observed in meningiomas (43), rat models of ischemia (44), hydrocephalus (45), and AQP4-knockdown brain (46). These results suggest that the ADC values correlate with AQP4 expression under certain pathological conditions, and ADC may be a surrogate marker of AQP4 expression. Based on this, we suggest that, similarly, the ADC of metastatic tumors may also reflect AQP4 expression.

In the glymphatic pathway, a periarterial influx of CSF is balanced by the perivenous efflux of interstitial fluid (47) under normal physiological conditions. The growth of metastases may disrupt this balance and result in the accumulation of interstitial fluid, *i*.*e*., PTBE. The inverse relationship between the ALPS index and the PTBE volume in our study suggests that a higher glymphatic function may facilitate interstitial fluid clearance and reduce or even prevent PTBE. As stated above, insufficient glymphatic function for interstitial fluid clearance may contribute to PTBE formation. A similar inverse relationship was observed in meningiomas, and glymphatic dysfunction was proposed to be the cause of PTBE (30).

In mice harboring gliomas and melanomas, glymphatic function is increased to reduce PTBE by remodeling of meningeal lymphatic vessels (MLVs), which are downstream of the glymphatic system (8). In mice with defective MLVs, impaired drainage of brain parenchymal interstitial fluid aggravates PTBE. We speculate that the brain metastases with higher ALPS index in our study may have greater remodeling of MLVs, which facilitates the efflux of interstitial fluid from the brain parenchyma. Alternatively, metastases with a higher ALPS index may have more glymphatic reserve capacity, which serves to relieve PTBE. To the best of our knowledge, no human studies have reported the inverse relationship between PTBE volume of metastases and interstitial fluid clearance in glymphatic system.

There are limitations to our study. First, the diffusion signal measured in clinical settings reflects overall changes in water mobility associated with many processes occurring at scales much smaller than typical MRI voxels. Therefore, we cannot definitely state that the ALPS index is a measure of glymphatic function, and the changes of tumor ADC were due to the expression of AQP4 and fluid volume increase in the tumor interstitium. The validation of ALPS index as a quantitative tool for measurement of glymphatic function is currently impeded by the invasiveness of evaluation (*e*.*g*., intrathecal contrast medium injection). Despite that, the potential of ALPS index to identify altered glymphatic function has been demonstrated in many neurological conditions. Second, our study is a snapshot in time and does not include longitudinal data on the temporal changes of ALPS index and PTBE volume following treatment. These pieces of information would be helpful to further establish the role of the glymphatic system in PTBE formation.

## Conclusions

In conclusion, metastases with higher tumor ADC and lower ALPS index were associated with larger peritumoral brain edema volumes. The higher tumor ADC may be related to increased periarterial water influx into the tumor interstitium, while the lower ALPS index may indicate insufficient fluid clearance. The changes in both tumor ADC and ALPS index may imply glymphatic dysfunction, which is, at least, partially responsible for peritumoral brain edema formation.

## Data Availability Statement

The raw data supporting the conclusions of this article will be made available by the authors, without undue reservation.

## Ethics Statement

The studies involving human participants were reviewed and approved by Chang Gung Medical Foundation Institutional Review Board. Written informed consent for participation was not required for this study in accordance with the national legislation and the institutional requirements.

## Author Contributions

CT, TS, and MC contributed to conception and design of the study. CT and TS contributed to the acquisition and analysis of data. CT and MC contributed to the drafting of the text and the preparation of the figures. The first draft of the manuscript was written by CT. All authors read and approved the final manuscript.

## Funding

This study was funded by the National Science Council Taiwan (NSC101-2314-B-182-084 to CT).

## Conflict of Interest

The authors declare that the research was conducted in the absence of any commercial or financial relationships that could be construed as a potential conflict of interest.

## Publisher’s Note

All claims expressed in this article are solely those of the authors and do not necessarily represent those of their affiliated organizations, or those of the publisher, the editors and the reviewers. Any product that may be evaluated in this article, or claim that may be made by its manufacturer, is not guaranteed or endorsed by the publisher.
